# Cardiovascular risk factors and frontotemporal dementia: a case–control study

**DOI:** 10.1186/2047-9158-3-13

**Published:** 2014-06-21

**Authors:** Angel Golimstok, Nuria Cámpora, Juan I Rojas, María C Fernandez, Cristina Elizondo, Enrique Soriano, Edgardo Cristiano

**Affiliations:** 1 Cognitive and Behavior Unit, Department of Neurology, Hospital Italiano de Buenos Aires, Perón 4272, 1411 Buenos Aires, Argentina; 2 Department of Neurology, Hospital Italiano de Buenos Aires, Buenos Aires, Argentina; 3 Epidemiology Department, Hospital Italiano de Buenos Aires, Buenos Aires, Argentina

**Keywords:** Cardiovascular risk factors, Frontotemporal dementia, Dementia, FTD, Diabetes, DBT, DM

## Abstract

Cardiovascular risk factors (CRF) were widely described as related to dementia. There are very few studies regarding this association in FTD. The objective of the study was to compare the frequency of CRF in our population with FTD and controls. 100 consecutive subjects with FTD diagnosis according to Lund-Manchester clinical criteria and 200 controls matched by age and sex were included between January 2003 to February 2007 at the Cognitive and Behavior Unit of Hospital Italiano de Buenos Aires. Clinical evaluation, laboratory tests, brain images (CT/MRI), neuropsychological and neuropsychiatric assessment were performed. Multiple regression analysis was performed to analyze the association in CRF between FTD patients vs. controls. The mean age in FTD was 69.7 ± 0.9 vs. 70.1 ± 0.8 in controls (p 0.12). No difference in gender was observed between cases and controls. No differences were identified between patients and controls regarding hypertension (HTA) (65% vs. 67,3% *p* 0.44); dyslipidemia (57% vs. 54.7% *p* 0.74); obesity (39% vs. 27.6% *p* 0.14) and hypothyroidism (26% vs. 17.1% *p* 0.1). A significant difference was observed for Diabetes Mellitus (39% vs. 22.6% *p* 0.001). In our population, Diabetes Mellitus was associated as an independent risk factor for FTD. To our knowledge this is the first report in which CRF were evaluated prospectively in FTD patients. More studies are needed to confirm this finding in larger populations.

## Introduction

Frontotemporal dementia (FTD) is a broad descriptive term referring to several distinct clinical syndromes characterized by progressive neurologic deterioration with prominent behavioral and language impairment [1,2]. There are three classic clinical syndromes: behavioral variant frontotemporal lobar degeneration (bvFTLD), semantic dementia (a fluent aphasia with loss of word meaning), and progressive non-fluent aphasia (a disorder typified by effortful, non-fluent speech). Frontotemporal dementia (FTD) accounts for 5–15% of all dementia and is the second commonest cause in the presenile age group [3,4]. About 20–40% of FTD cases are familial in series from specialist referral centers [5-8]. Among the FTD syndromes, bvFTLD is the most heritable, and semantic dementia the least heritable, perhaps accounting for recent evidence suggesting that a high proportion of patients with semantic dementia present over the age of 65 years [9]. A knowledge of risk factors for FTD may provide clues to the underlying pathophysiology. There is increasing interest in genetic factors that may predispose to the disease, and five genetic loci for causal mutations have been identified, all showing 100% penetrance. However, approximately 60% of patients with FTD have no family history of dementia and are considered to be sporadic cases. Prospective studies evaluating non-genetic risk factors for this condition are lacking.

A large number of factors have been associated with increased risk of dementia in general, and cardiovascular risk factors are the most consistently reported. A history of diabetes, hypertension, smoking, obesity, and dyslipidemia have all been found to increase risk. Although these risk factors are well studied in Alzheimer’s Disease (AD), Vascular Dementia (VD) and Lewy Body Disease (LBD) [10-16], very few reports are found in FTD.

We therefore undertook a case–control study to analyze the association of cardiovascular risk factors and FTD.

## Methods

### Site

This study was conducted at the Italian Hospital Medical Care Program (IHMCP) in Buenos Aires, Argentina with approval from the institutional Review Board of the IHMCP research committee. IHMCP provides comprehensive medical and health services to over 150,000 members primarily located in the urban areas around the Autonomous City of Buenos Aires, Argentina. The IHMCP population characteristics are closely representative of the metropolitan population of the Autonomous City of Buenos Aires, as demonstrated by 2001 census data in a series of socio-economic categories (Table 1). The period of the study was conducted from 2003 through 2007. Patients and controls were analyzed after informed consent was signed. In demented patients, researchers ensure that patients fully understand and appreciate the consequences of their participation throughout the course of the study. When a demented patient was not able to make informed decisions, the researchers ensured that the substitute decision maker (a direct family member) made the choice regarding patient wishes. Patients with dementia and controls were recruited from the membership of the IHMCP, a large prepaid health maintenance organization model.

**Table 1:** **Socioeconomic level and ethnic origin of inhabitants of the Autonomous City of Buenos Aires and IHMCP affiliates**, **based on the 2001 Argentinean census**

	**City of Buenos Aires (%)**	**IHMCP (%)**
**Upper class**	10	5
**Upper middle class**	16	19.4
**Middle class**	30	37.5
**Lower middle class**	21	25.6
**Lower class**	17	12.5
**Poor**	13	0
**Total**	100	100
**Ethnic origin**		
Caucasian	92	95.5
Asian	4	2
African American	1	0.5
Mestizos (a)	3	2

IHMCP, Italian Hospital Medical Care Program. (a) Mestizos (Spanish term used to designate people of mixed European and Amerindian ancestry living in the region of Latin America).

### Patients

Our study included patients who met Lund and Manchester criteria [17]. All patients were evaluated and diagnosed by a trained neurologist. Clinical evaluation, laboratory tests, brain images (CT/MRI) and neuropsychological-neuropsychiatric assessment were performed. Routine clinical investigations were conducted to exclude reversible causes of dementia. Patients were excluded if formal examination showed evidence of any other brain disorder, physical and/or mental illness that contributed considerably to the clinical picture. Patient selection was strictly consecutive and included all prevalent cases in the center who met previous criteria.

### Controls

Controls were matched to patients with FTD by sex, age and geographic area of residence. For each patient, we identified two people from the same general practice list of the same sex and age. If a potential control was ineligible, we approached the next closest in age. Controls were never duplicated. Records of potential controls were reviewed by a neurologist to exclude those controls in which the presence of dementia of any type or any other neurological disease was suspected before or during the index year (year of diagnosis of dementia in the matched case). The list of the entire population from which potential controls were randomly drawn was provided by the record database system of the IHMCP epidemiological center, and control subjects were selected for cases using a statistical program.

### Ascertainment of cardiovascular risk factors

In order to assess and compare the frequency of cardiovascular risk factors in our population with FTD and controls, the medical records obtained during a 4-year period (1 January 2003 to 28 February 2007) at IHMCP were examined. We confined the cardiovascular risk factor assessment to: age, sex, hypertension (HTA) (in which hypertension was defined as diastolic blood pressure >90 mmHg and systolic blood pressure >140 mmHg. The diagnosis was based on medical history, current treatment and results of direct measurements performed on three different occasions during the study), dyslipidemia (TG ≥ 150 mg/dl. HDL-C < 40 mg/dL for males, < 50 mg/dL for females and LDL cholesterol >130 mg/dl), obesity (defined when body mass index, calculated as the ratio between weight and squared height, was >30 kg/m2.), osteoporosis and hypothyroidism (TSH >4.5 mU/L) (by possible association with dyslipidemia). CVRF were considered according to CAIDE study data [18] and Diabetes (DM) (fasting plasma glucose level ≥ 7.0 mmol/l or 126 mg/dl) according to the Rotterdam study [19].

### Statistical analyses

Associations for potential cardiovascular risk factors were assessed with conditional logistic regression analyses comparing FTD and control subjects. This was done in order to identify the collection of features that displayed differences between study groups while controlling for the effects of the others. Stata 10.1 was used for this analysis.

Ethics approval was obtained from the Ethics Committee of Hospital Italiano de Buenos Aires, Argentina. Informed consent was obtained from each case and control included.

## Results

During the study period, a total of 100 cases and 200 controls were included. The mean age in FTD cases was 69.7 ± 0.9 vs. 70.1 ± 0.8 in controls (p 0.12). A total of 75% of the population was of female sex in controls vs. 68% in cases included. No sex difference was observed between cases and controls, as was expected . When the included population from the IHMCP was compared with the socioeconomic and ethnic origin data from the City of Buenos Aires, no significant differences were observed. A total of 65% of cases had hypertension, 39% obesity and 39% fulfil diagnosis criteria for diabetes. Baseline characteristics of patients included are displayed in Table 2. When cases and controls were compared, no association in hypertension (HTN) (65% vs. 67.3% p 0.44), dyslipidemia (57% vs. 54.7% p 0.74), obesity (39% vs. 27.6% p 0.14) and hypothyroidism (26% vs. 17.1% p 0.1) were found in the logistic regression analyses performed. However, we observed significant association in the logistic regression analysis in Diabetes Mellitus type II (DM2) between cases and controls (39% vs. 22.6% p = 0.001) (Table 3).

**Table 2:** Baseline Characteristics of included patients*

**Variable**	**Controls**	**Cases**
	**N = 200**	**N = 100**
**Female sex n (%)**	150 (75)	68 (68)
**Mean age (SD), years**	70.1 ± 0.8	69.7 ± 0.9
**DM2 N (%)**	45 (22.6)	39 (39)
**HTN N (%)**	134 (67.3%)	65 (65%)
**Obesity N (%)**	55 (27.6%)	39(39%)
**Dyslipidemia N (%)**	109 (54.7%)	57 (57%)
**Hypothyroidism N (%)**	34 (17.1)	34 (26)
**Osteoporosis N (%)**	40 (20.1%)	21 (21%)

*Data shown as baseline descriptive variables. Comparisons between groups were made by logistic regression analyses.

DM: Diabetes mellitus, HTN: hypertension.

**Table 3:** Logistic regression analyses

**Variable**	**P**	**OR (95% CI)**
**DM2**	**0.001**	**4.8 (2.1-6.5)**
HTN	0.44	1.3 (0.8-2.1)
Obesity	0.14	1.2 (0.6-1.8)
Dyslipidemia	0.74	1.5 (0.5-2.2)
Hypothyroidism	0.10	1.8 (0.9-3.1)
Osteoporosis	0.36	1.3 (0.7-2.5)

DM: Diabetes mellitus, HTN: hypertension.

## Discussion

In this study, we examined the frequency of 6 possible risk factors associated with the likelihood of FTD. Among the CVRF we did not include tobacco, as it was very difficult to verify the data in our control group records. A clinical diagnosis of FTD was associated with a greater likelihood of having a history of DM2. This is a very significant finding in our population whereas in Latin America, diabetes is a highly prevalent disease in over 45 years, with higher percentages of cases than the rest of the world [20]. In two previous retrospective case–control studies the prevalence of this antecedent was higher in patients with FTD, but the multivariate analysis showed there were no statistically significant differences [5,21]. The explanation for this association remains unclear, but several findings from clinical and experimental research encourage the formulation of hypotheses about the underlying mechanisms. Previous prospective, large, population-based cohort studies have found that diabetes is associated with an increased risk of cognitive decline and dementia [19,22,23].

The principal finding of these studies was that diabetes is associated with a 50-100% increase in risk of AD and of dementia overall and a 100-150% increased risk of vascular dementia. However, limitations in clinical diagnostic criteria do not allow the confirmation of a differential effect on dementia subtype [24].

Type 3 diabetes mellitus (DM3) was described as corresponding to a chronic insulin resistance plus insulin deficiency state largely confined to the brain that can overlap with DM2 [25]. As It has been proposed that DM3 represents a major pathogenic mechanism of AD neurodegeneration [25,26], in the last years have been made several studies demonstrating the biological substrate of this mechanism [27-29].

As was expected, such studies have not been performed in FTD because the association with DM has not been reported so far.

Areas of active research are the direct effects of hyperinsulinemia and insulin resistance in the brain. Abnormal insulin sensitivity was found to be associated to deficits in speech production (verbal fluency), reduction of gray matter volume in the temporal lobe brain regions related to language function and lower total brain size as well [23].

As language processing is usually impaired in patients with FTD [30,31], these findings should be analyzed. There is evidence that Central Nervous System (CNS) insulin signaling enhances synaptic long-term potentiation [32], modulates the action of neurotransmitters involved in behavior and cognitive processing (e.g., norepinephrine and dopamine) [33] and attenuates cortisol secretion [34]. Thus, we can infer that more than one mechanism is involved in the pathophysiology of these changes and that DM2 may represent a metabolic state in which neuroprotective and neuromodulatory effects of insulin in the CNS are disrupted. This may be particularly true in FTD because the process of neurodegeneration takes place in brain regions with high densities of insulin receptors that are sensitive to changes in CNS insulin signaling, such as in the temporal lobes [35]. Analogous findings related to similar pathologies demonstrating reduced cerebral glucose metabolic rate in frontal, parietotemporal, and cingulate brain regions support these concepts [36]. Furthermore, chronic hyperglycemia, the hallmark of DM has been shown to accelerate formations of advanced glycation end products (AGEs) that can induce tau hyperphosphorylation which, in turn, impairs synapse and memory through RAGE-mediated GSK-3 activation [37]. Tau hyperphosphorylation may be an important event in the process leading to tau intracellular aggregation and neuronal cell death in tauopathies that constitute a significant percentage of cases of FTD [38].

Supporting this concept, it is known that insulin exerts its effect through a receptor, a heterotetramer (α2β2) with tyrosine kinase activity in its intracellular portion. Joining insulin extracellular α subunits induces a change in the conformation of β subunits, which initiates a series of phosphorylations and activates, among others, the path of PI3-K/Akt. Activation of PKB or Akt deactivates glycogen synthase kinase-3 (GSK-3) [39-41].

Another piece of interesting data that may help to understand the meaning of the association between DM2 and FTD are the studies reporting brain atrophy in DM2 patients mainly exhibiting gray and white matter atrophy in right temporal lobe, and this finding bears out that DM2 could lead to subtle diabetic brain structural changes in patients without dementia or macrovascular complications [42]. This again corroborates findings in adults with DM2, where prefrontal volume reductions and global cerebral atrophy have been reported. Patients with diabetes, both with and without depression, had smaller total brain gray matter volumes when compared with the control subjects after controlling for age, intracranial volume and years of education. This group also had smaller gray matter volumes in the anterior cingulate and orbitofrontal regions when compared with the controls after additionally controlling for total gray matter volume. The depressed and nondepressed diabetic groups did not differ in any neuroimaging measure in this series of patients reported [43].

Another report showed that greater insulin resistance as indexed by HOMA-IR was associated with an AD-like pattern of reduced cerebral metabolic rate of glucose in frontal, temporal-parietal, and cingulate regions in adults with pre-diabetes or DM2 [36].

Interestingly, a similar pattern of brain regional glucose metabolism has been recently described in FTD as well [44]. Obesity was reported as related to brain atrophy, cognitive deficit [45-47] and as a risk factor for other types of dementia [48]. However, in our study we found no statistically significant differences between groups in the multivariate analysis. So, we suggest that obesity was not an independent risk factor for FTD, in this population. As in previous reports [21,49], our study showed that the percentage of hypothyroidism was higher in cases than in controls, but the difference was not significant in the multivariate analysis. Mental studies have shown that splicing of juvenile and adult tau mRNA variants is regulated by thyroid hormone. As shown by the in situ hybridization experiments, thyroid hormone seems to regulate the levels of tau mRNA in the cerebellum by changing the rate of migration of the granule cell. Another effect of this hormone on tau expression is to modify the timing of the splicing mechanism that during development allows differential selection of exons present in the tau gene. It remains to be determined whether the effects of thyroid hormone on cell migration and on the splicing mechanism are related. It is also not clear whether the post-transcriptional effect of thyroid hormone on the splicing mechanism is direct or, more probably, mediated by gene expression. It is widely accepted that the cellular actions of thyroid hormone are mediated by nuclear receptors that bind to thyroid hormone response elements associated with target genes and stimulate or inhibit expression of these genes. The data reported herein suggest that, at least for the production of various tau protein isoforms during brain development, the splicing mechanism is regulated by thyroid hormone. Even if this effect of thyroid hormone is indirect it may explain the impairment in neurite outgrowth induced by thyroid hormone deficiency in the newborn [50]. The cause of one of the most common syndromes of frontotemporal dementia are the tauopathies, and thyroid hormone level abnormalities in frontotemporal dementia were found to be frequent (38%) in a previous study [51]. As thyroid disorders may be related to FTD, further studies to elucidate the mechanisms of this association are needed. The other factors studied such as hypertension, dyslipidemia and osteoporosis were not shown to be risk factors for FTD, which is consistent with results of previous reports. We include osteoporosis among risk factors because it has been reported previously that the vitamin D deficiency is a common finding in dementia [52].

The strength of our study, with a considerable number of patients and controls, was that patients were fully studied and followed over time to verify the diagnosis in their evolution. Unfortunately, we have no pathology of our cases, and controls were not studied directly but through examination of medical records. This last limitation prevented us to compare the images and cognitive scores between the two groups.

Our findings open the door to prospective studies with pathology that distinguish between different variants included in the diagnosis of FTD and that examine other factors not analyzed in the present study.

## Competing interest

A. Golimstok, C. Fernández, N. Cámpora, C Elizondo and E. Soriano declares no conflict of interest.

Juan Ignacio Rojas has received honoraria from Novartis as a scientific advisor. He has received travel grants and attended courses and conferences on behalf of Merck-Serono Argentina, Novartis Argentina.

Edgardo Cristiano has received fees for consultations as a scientific advisory board member and for travel to meetings, conferences and clinical trials of the following companies: Avanir, Bayer, Biogen, Merck, Novartis and Teva.

## Authors’ contributions

AG, NC, MCF and EC design and study analysis. JIR, CE, ES statistical design and analysis. All authors read and approved the final manuscript.
